# Application and Experimental Substantiation of the Radioecological Model for Prediction in Behavior ^90^Sr in Cultivated Soil-Crop System: A Case Study of Two Experimental Agricultural Fields

**DOI:** 10.3390/plants13131798

**Published:** 2024-06-29

**Authors:** Nataša B. Sarap, Marko Ž. Daković, Ivica Djalovic, Željko Dolijanović, P.V. Vara Prasad, Marija M. Janković

**Affiliations:** 1 Vinča Institute of Nuclear Sciences, National Institute of the Republic of Serbia, Department of Radiation and Environmental Protection, University of Belgrade, Mike Petrovića Alasa 12–14, 11001 Belgrade, Serbia; marijam@vin.bg.ac.rs; 2 Faculty of Physical Chemistry, Department of Radiochemistry and Nuclear Chemistry, University of Belgrade, Studentski trg 12–16, 11000 Belgrade, Serbia; marko@ffh.bg.ac.rs; 3 Institute of Field and Vegetable Crops, National Institute of the Republic of Serbia, Maksima Gorkog 30, 21000 Novi Sad, Serbia; maizescience@yahoo.com; 4 Faculty of Agriculture, Department of Agroecology and Agrotechnics, University of Belgrade, Nemanjina 6, 11080 Belgrade-Zemun, Serbia; dolijan@agrif.bg.ac.rs; 5 Department of Agronomy, Kansas State University, 108 Waters Hall, 1603 Old Claflin Place, Manhattan, KS 66506, USA; vara@ksu.edu

**Keywords:** radiostrontium, soil–crop pollution, transfer model

## Abstract

The radioactive fission product ^90^Sr has a sufficient half-life (28.8 years) to be detected long after its appearance in the environment. After its uptake into the soil-edible plant system, it enters the food chain and represents a potential source of contamination that threatens human health. Due to these facts, tracking the distribution of the artificial radionuclide ^90^Sr in the soil–edible plant system is a subject of intense research. The tracking of the ^90^Sr radionuclide distribution in the soil profile, as well as in the crops on the long-term experimental fields was carried out using beta radiation spectrometry. The radiochemical analytical method was used to analyze the ^90^Sr content in cultivated soil and crops. The conducted study focused on the experimental substantiation of the developed model for predicting the behavior of ^90^Sr in the cultivated soil–crop system. The results of using the applied radioecological model for the transfer of ^90^Sr from the soil to the above-ground part of crops showed a relatively good agreement with the experimentally determined values of the soil–crop transfer factor, which indicates that the used model can be successfully applied for the prediction of the behavior of ^90^Sr in the soil–soil solution–crop system.

## 1. Introduction

Human population may be exposed to radiation emitted from various radioactive sources [1]. In addition to the risk released during the testing and use of nuclear weapons, accidents in nuclear power plants and in the nuclear fuel reprocessing industry, applications in medicine or in scientific research can also contribute to an increase in the concentration of some radionuclides in the environment. Since the middle of the last century, particularly since the accident at the Chernobyl nuclear power plant in 1986, there has been an increase in artificial radionuclide content in the environment.

The radioactive strontium isotope ^90^Sr is present in measurable quantities in the environment worldwide as a result of intentional or unintentional releases, either from atmospheric nuclear weapons tests or at the local level through authorized or unauthorized releases from nuclear facilities and nuclear accidents [2]. Radionuclide ^90^Sr can be regarded as a highly hazardous anthropogenic radionuclide belonging to the group of long-lived fission products (half-life is 28.8 years). This radionuclide emits a β-particle of 546 keV, from which ^90^Y is produced, a hard β-emitter (half-life is 64.2 h and the energy of the emitted β-particle is 2.28 MeV), which reaches secular activity equilibrium in 14 days [3]. Strontium as an alkaline earth metal (in this case the radionuclide ^90^Sr) follows the chemical and metabolic pathways of calcium in living organisms. Humans can be contaminated with ^90^Sr by inhalation (dust), ingestion (food, mainly milk and dairy products) or direct contact via transcutaneous absorption, but the most important exposure pathway is ingestion [4]. The radionuclide ^90^Sr is mainly incorporated into bone tissue in the form of hydroxyapatite and belongs to the group of osteotropic radionuclides [5]. Due to the fact that this radionuclide can potentially cause bone cancer (osteosarcoma), leukemia, and lung or skin cancer, it has been designated a “human carcinogen” [6].

The radionuclides dissolved in the rainwater enter the soil through the filtration process. These substances can exist in various forms in soil and different forces keep them bound to soil particles. Most radionuclides are trapped within the surface soil by strong sorption to soil minerals, such as clays and iron oxides, and their downward migration is hindered. Studies have shown that ^90^Sr is not easily leached from the soil even during heavy rainfall [5]. Furthermore, published data indicate that the ion exchange mechanism of ^90^Sr in soil is predominant and that the amount in soil solutions is relatively high compared with other long-lived radionuclides such as ^137^Cs, ^241^Am or ^239^Pu [7].

The results of previous research suggest that the migration of radionuclides in soil depends on a number of environmental factors: the intensity of their deposition from the atmosphere (e.g., maturation rate and precipitation) and synergistic influences of natural conditions (vegetation, climate, relief, i.e., local terrain configuration). It depends in particular on the physico-chemical properties of the soil (e.g., organic matter content, adsorption complex properties, pH, mineralogical composition), the structure (mechanical composition, porosity), the moisture content (water content, groundwater) and the agrotechnical measures applied (drainage and degree of treatment, fertilization) [8].

With regard to the contamination of food with the radionuclide ^90^Sr, it is important to know the migration pathways under natural conditions and the extent to which plants can be contaminated with this fission product. There are two mechanisms that allow the uptake of ^90^Sr by crops: direct uptake by the above-ground part of the plant from the air, especially after fallout, to varying degrees, and indirect uptake from the soil through the root system of the plant [9]. Radionuclides present in the soil enter the plant roots in the same way as their stable counterparts. The uptake of long-lived radionuclides by a plant depends, to a large extent, on whether the radionuclide remains within the reach of the plant’s root system, on the extent to which it is chemically available, on the metabolic needs of the plant and on the physico-chemical properties of the soil. The transfer of ^90^Sr in the soil–plant ecosystem largely depends on the amount of solvated ^90^Sr^2+^ is available to the plant. The morphological and physiological characteristics of plant cultures (species, structure, growth, length of the growing season) are very important factors determining the degree of radionuclide uptake.

In radioecological studies to estimate the radiation dose received by a human being through the uptake of radionuclides into the body, the activity concentration ratio is inevitably used in the models as a quantifier of the transfer of radionuclides from one link in the food chain to another. In addition to assessing the radiation risk, knowledge of the value of this ratio can be used to standardize the value of the minimum detectable activities of radioactive contaminants in agricultural soil, to establish preventive measures for accidents and to suggest possible decontamination of agricultural soil through the cultivation of certain crops [10]. Among other things, data can be obtained on the basis of a theoretical explanation of the different uptake of elements that are not involved in the development of physiological and biochemical processes in plant cultures. This group includes the artificial radionuclide ^90^Sr, whose toxicology includes both radiation and chemical toxicity, which depend on several factors, complicating and exacerbating the adverse effects on human health due to exposure to a particular radionuclide [11,12].

Migration in the soil–plant system is primarily determined by the physico-chemical properties of the radionuclides and the soil, the biological properties of the plants and the agrotechnical measures applied during soil cultivation. For these reasons, the estimation of the activity concentration ratio is the subject of many studies [13,14,15,16,17,18,19,20,21]. Mathematical knowledge in the form of predictive models is used to simulate or represent the radioecological processes in the biosphere, as well as a number of very complex radiobiological processes in living organisms [22]. The models, which assess the radioecological effects on the environment, can help in the regular monitoring of the regulation of routine releases of radionuclides into the environment, as well as in the planning of necessary measures in the event of accidents and the prediction of health, economic and social effects. It is important to check the reliability of such predictions in comparison with the measured values in the environment or in comparison with the predictions of other models [22]. The prediction results can be improved by using parameters that have been experimentally determined for the analyzed system, such as the activity concentration ratio of radionuclides in the food chain. As the soil–plant system is an important part of terrestrial ecosystems, more data are needed on the role of this system in the transfer of radionuclides, especially because of the potential impact on the food chain. A better understanding of this transfer could help to quantify the main processes that influence the behavior of radionuclides within the said system, which is one of the most important scientific challenges [23]. For the prediction of radioecological processes in the biosphere, the distribution of certain activity concentrations as a function of time is crucial, while a number of other processes such as uptake, migration, translocation, etc., must also be considered. The study of the regularity of the migration of radioactive fission products in the food chain offers the possibility to assess with some accuracy the radiation situation in the area exposed to radioactive contamination. There are a number of dynamic models for predicting the extent of radionuclide content in the biological cycle of the food chain [24,25,26,27]. The complex process of ^90^Sr transfer from soil to plant is determined by a number of different physico-chemical and biological processes that can only be formalized in a mathematical model.

This study was dedicated to determining the ^90^Sr content and tracking its behavior in a cultivated soil–crop system in long-term experimental fields. The specific objectives were to verify the transfer of ^90^Sr within the studied system. Considering the general lack of data in this field and in general for the investigated soil types, especially for the territory of the Republic of Serbia, the present study provides new information on ^90^Sr transfer in the context of the soil (chernozem)–crop system.

## 2. Materials and Methods

### 2.1. Sample Collection and Preparation

Cultivated soil and crop samples were collected in the experimental agricultural farm “Radmilovac” in the Belgrade region (44°45′ N, 20°34′ E) and in the long–term experimental field “Rimski Šančevi” in the Novi Sad region (45°19′ N, 19°50′ E), both in the Republic of Serbia. Sampling was carried out three years in a row from 2013 to 2015, in spring or summer, depending on the crop type, as the crops were sampled at the stage of full maturity. The soil samples were sampled at the same time and from the same measurement points as the crops. Each year, samples were taken in triplicate from six plots in “Radmilovac” (located at an altitude of 143 m and part of a four-field crop rotation) and from ten plots in “Rimski Šančevi”(located at an altitude of 90 m and part of various crop rotations and continuous cultivation). The soil samples (leached chernozem at the “Radmilovac” site and chernozem at “Rimski Šančevi” site) were taken at a depth of (0–20) cm; this profile was chosen because it represents the root zone of the selected crops. The crop samples were sampled as a whole with all parts, from root to fruit. The sampling included cereals: alternative winter wheat species (Triticum durum, variety Durumko), common bread wheat (Triticum aestivum vulgare, variety Rapsodija) and the maize hybrid ZP SC 677 (*Zea mays* L.) at the “Radmilovac” site; cereals: bread wheat (*Triticum aestivum* L., variety NS 40S) and the maize hybrid NS 6010 (*Zea mays* L.); as well as commercial crops: soybean (*Glycine max* L. Merr, variety Venera) and winter rapeseed (*Brassica napus* L., variety Zlatna) at the “Rimski Šančevi” site.

Sample preparation and analysis of ^90^Sr were carried out at the Department of Radiation and Environmental Protection of the Vinča Institute of Nuclear Sciences. The collected soil samples were oven-dried at 105 °C for 24 h and then crushed, sieved through a stainless-steel sieve (sieve size 250 μm) and homogenized. In addition, the samples were ashed for 17 h at 500 °C. The collected crop samples were washed with distilled water and soaked in 0.1 M HCl for several hours to remove soil residues and mineral oxides from the surface; they were divided into parts manually: winter wheat and rapeseed into the root and the rest of the plant (stem with grains), while maize and soybean were separated into individual plant organs (root, stem, leaf and grain). The crop parts were air-dried at room temperature for three weeks and then ashed at 450 °C for 24 h.

### 2.2. Analysis of the Physico-Chemical Properties of Soil

Before the preparation of soil samples for analysis of ^90^Sr, the physico-chemical properties were analyzed by standard methods at the Faculty of Agriculture of the University of Belgrade. To determine the mechanical composition of the soil, the pipette method was used [28]. Hygroscopic humidity was determined by gravimetric water loss from soil samples, the masses of which were measured before drying at 105 °C. The density or volume mass, in the natural undisturbed state of the soil, is determined by Kopecky cylinders, with a volume of 100 mL [29]. Active and substitutional acidity in the soil were determined electrometrically using a pH-meter with a double combined electrode, namely, active acidity in the ratio of soil and distilled water 1:2.5, and substitutional acidity in the same ratio of soil and 1M KCl [30]. The quantitative content of CaCO_3_ was determined volumetrically by measuring the volume of released CO_2_, using Scheibler’s calcimeter [31]. The humus content was determined by Tyurin’s method [32]. The content of organic carbon was determined by titration with (NH_4_)_2_Fe(SO_4_)_2_·6H_2_O, after digestion of samples with dichromate sulfuric acid solution [33].

### 2.3. Radiochemical Analysis and Measurement of Radioactivity

Radiochemical analysis was performed at the Department of Radiation and Environmental Protection of the Vinča Institute of Nuclear Sciences. The laboratory for radiation measurements in this department is accredited according to ISO/IEC 17025. The radionuclide ^90^Sr was determined by the radiochemical separation of ^90^Y from the sample after the radioactive equilibrium was established. Detailed information on the method used has been described by Sarap et al. [34]. Beta spectrometry with the Thermo Eberline FHT 770T gas flow proportional counter (ESM Eberline Instruments GmbH, Erlangen, Germany) was used to detect and analyze the ^90^Sr activity concentration in the investigated samples. Calibration of the gas flow proportional counter for beta spectrometry was performed using a point calibration source for ^90^Sr (9031–OL–335/11, issued by the Czech Metrology Institute) traceable to BIPM (International Bureau of Weights and Measures). The counting efficiency for beta particles is 35%. The time interval for sample counting was 5400 s (in three replicate series).

The ^90^Sr activity concentration (A_Sr-90_) in the soil and crop samples, expressed in Bq/kg, was calculated using the following equation:

(1) ASr-90=(R-R0)·eln2T12·tε·ηs·η·m

where R is the count rate of the measured sample (1/s), R_0_ is the background count rate (1/s), t is the time elapsed since ^90^Y separation (h), T_1/2_ is the half-life of ^90^Y (h),ε is the beta counting efficiency, η_S_ is the chemical yield for the measured sample, η is the yield factor of the method used and m is the mass of the soil and crop sample (kg).

The measurement uncertainty of the determination of the ^90^Sr activity concentration (∆(A_Sr-90_)) was calculated using the following equation:

(2) $$\Delta (A_{Sr-90})=A_{Sr-90}\cdot \sqrt{{\left(\delta R\right)}^{2}+{\left(\delta T_{1/2}\right)}^{2}+{\left(u\left(\varepsilon \right)\right)}^{2}+{\left(\delta \eta_{s}\right)}^{2}+{\left(\delta \eta \right)}^{2}+{\left(\delta m\right)}^{2}}$$

where δR is the relative measurement uncertainty of the count rate defined by the software and expressed as the relative standard deviation, δT_1/2_ is the relative measurement uncertainty of the factor in the exponent of Equation (1), which is defined as (ln2·t·ΔT_1/2_/(T_1/2_)^2^), u(ɛ) is the relative measurement uncertainty of the beta-counting efficiency, δη_S_ is the relative measurement uncertainty of the chemical yield for the measured sample, while δη is the relative measurement uncertainty of the method yield factor and δm is the relative measurement uncertainty of the mass measurement.

The minimum detectable activity (MDA) is calculated according to Equation (3):

(3) $$MDA=\frac{2.71+4.65\cdot \sqrt{R_{0}\cdot t_{0}}}{\varepsilon \cdot \eta_{s}\cdot m\cdot t_{s}}$$

where t_0_ is the background measurement time (s) and t_S_ is the sample measurement time (s), while R_0_, ε, η_S_ and m have the same meaning as in Equation (1).

With a counting time of 5400 s and a beta counting efficiency of 35%, the MDA was on average 0.33 Bq/kg per soil sample and 0.04 Bq/kg per crop sample.

### 2.4. Estimation of the Transfer Factor

The estimation of the transfer factor is one of the most important radioecological parameters or parameters of radiation protection. The transfer factor (TF) as a quantitative measure of the transfer and accumulation of ^90^Sr radionuclide in the agricultural soil–crop system is calculated according to the following equation:

(4) $$TF=\frac{A_{Sr-90,crop}}{A_{Sr-90,soil}}$$

where A_Sr-90, crop_ is the measured activity concentration of ^90^Sr in the crop sample (Bq/kg dry mass) and A_Sr-90, soil_ is the measured activity concentration of ^90^Sr in the soil sample (Bq/kg dry mass).

### 2.5. Statistical Evaluation of the Measurement Results

The statistical evaluation of the measurement results was carried out using linear correlation analysis and analysis of variance (ANOVA) to determine the existence of significant differences in the chemical composition and the ^90^Sr activity concentration in the soil and in the crops for the aforementioned experimental agricultural farms. The correction for multiple comparisons was performed using Tukey’s HSD test with a significance level of 0.05. The model considers all variables and their mutual influence, which cannot be assessed in any other way. The initial hypothesis to be tested is verified by performing the F-test. The calculated F-value is compared with the critical value. If it is greater than the critical value, the initial hypothesis (null hypothesis) is rejected with a risk of less than 5% (*p* < 0.05), i.e.*,* the mean values differ significantly, and if the calculated value is less than the critical value, the null hypothesis is retained with a risk of more than 5% (*p* > 0.05). The resulting set, which determines the deviations, is determined by comparing the mean values in an ascending series and comparing the differences in neighboring values with the smallest significant difference.

### 2.6. Mathematical Modeling of ^90^Sr Transfer in the Agricultural Soil–Crop System

**Figure 1:**
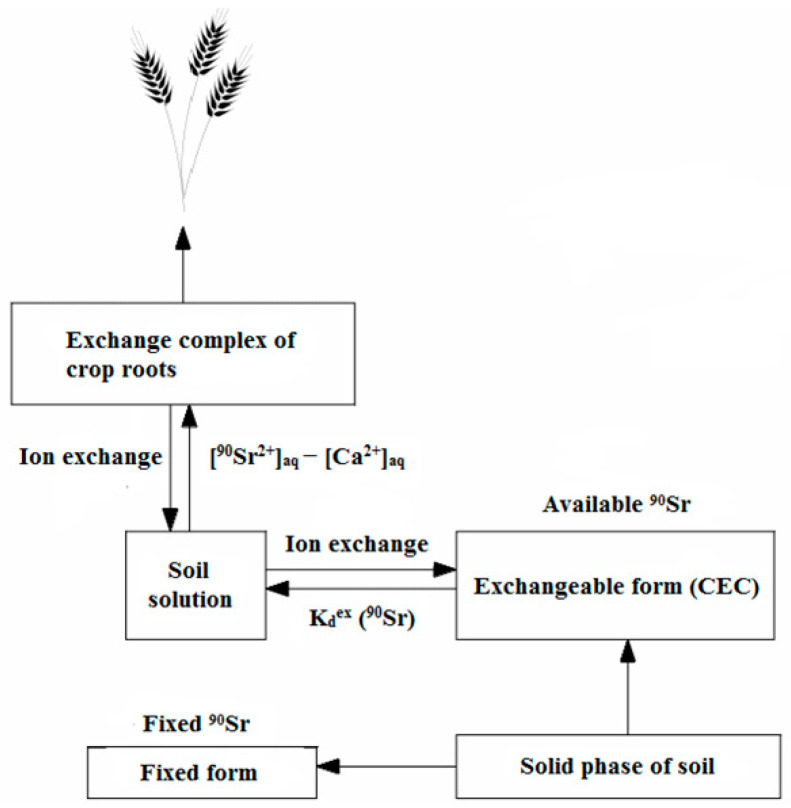
Schematic representation of the elements of the mathematical model of ^90^Sr transfer in the solid phase of the soil–soil solution–plant system. The figure is taken from the reference [7] and modified.

In this study, the model of ^90^Sr transfer in the solid phase of the soil–soil solution–plant system developed by Maskalchuk et al. [7], was applied. Within the above-mentioned paper, the basic assumptions of the mathematical model simulating the transfer of ^90^Sr from the soil to the above-ground part of the plant were described in detail based on the relevant assumptions. A schematic representation of the elements of the mathematical model of ^90^Sr transfer in the solid phase of the soil–soil solution–plant system is shown in Figure 1. In Figure 1, K_d_^ex^ represents the exchange partition coefficient of ^90^Sr between the solid and liquid phases of the soils.

Based on the developed model, the transfer of ^90^Sr from the soil into the plants can be determined using the following relationship [7]:

(5) TF=ASr-90,cropASr-90,soil=k·αexKc(Sr 2+90/Ca2+)·CEC

where k characterizes the efficiency of ^90^Sr uptake by a plant, α_ex_ is the fraction of exchangeable ^90^Sr in the soil (%), K_c_ (^90^Sr^2+^/Ca^2+^) is the exchange selectivity factor for the Sr/Ca cation pair in the solid phase of the soil and CEC is the cation exchange capacity (cmol/kg). From the experimental data, the model requires knowledge of the ^90^Sr activity concentration in the above-ground part of the plant and in the root system as input parameters. In this context, the application and the experimental substantiation of the model for the prediction of the behavior of ^90^Sr in a cultivated soil–crop system are carried out.

## 3. Results and Discussion

### 3.1. ^90^Sr Activity Concentration in Investigated Agricultural Soil and Crop Samples

The distribution of ^90^Sr radionuclide in agricultural soil and crops using beta radiation spectrometry in the spring or summer season during the three-year study period is presented. The values of ^90^Sr activity concentrations in the soil ((0–20) cm depth, around the crop root) of both experimental fields are presented in Table 1. The measurement results are expressed as Bq/kg dry mass with a confidence level of 95% (k = 2). The data from Table 1 show that the activity concentrations of ^90^Sr for the soil of the experimental field “Rimski Šančevi” are lower than the values for the experimental field “Radmilovac”. This could be explained by different geochemical factors of the soil and climatic phenomena, which can cause a difference in the cumulative deposition of ^90^Sr in the soil by a factor of 10 in the same latitude interval [35].

The values of ^90^Sr activity concentrations in the analyzed crop samples for three years of tracking in two experimental fields are summarized in Table 2. The measurement results were corrected according to the date of sampling and are expressed in Bq/kg with the confidence level of 95% (k = 2). Due to the double crop rotation on the experimental field “Radmilovac” and the double and triple crop rotation on the experimental field “Rimski Šančevi”, Table 2 contains the results of ^90^Sr activity concentration for each crop type during the entire study period.

From the values presented in Table 2, it can be seen that the activity concentrations in winter wheat at the same sampling sites on the experimental field “Radmilovac” are lower in the third year of sampling, which is consistent with the decreasing trend of ^90^Sr activity concentration during the years of soil investigation. There were differences in the distribution of ^90^Sr activity concentrations across crops. In winter wheat and rapeseed, most of the activity concentration remained in the root, and a much smaller part was transferred to the rest of the plant. In maize and soybean, most of the activity concentration also remained in the root, with redistribution to the individual plant organs in the following order: root > leaf > stem > grain. In addition, winter wheat took up more ^90^Sr than rapeseed, while soybean took up more than maize. The abovementioned could be due to the fact that legumes ensure the retention of strontium in the soil solution due to the specific mechanism of nitrogen uptake and the enrichment of the soil with nitrates. A comparison of the ^90^Sr activity concentration in the same crops at both experimental fields shows that the path of transfer of this radionuclide in the plant organs is the same, but the average values are slightly higher in winter wheat and maize on the experimental field “Radmilovac”.

### 3.2. Physico-Chemical Properties of the Soil

In order to investigate any relations between the ^90^Sr activity concentration and the main soil properties, several physical and chemical properties of the soil were determined. The results of the determination of the physical properties of the studied soil at two experimental fields at a 20 cm depth are presented in Table 3.

Based on the granulometric composition of the soil, it was noticed that the examined soil of both experimental fields belongs to loam characterized by a powdery-clay texture, with a low sand content and an extremely high, but favorable, powder content and slightly lower clay content. According to the soil classification [36], which takes into account the aggregate fraction of sand and clay, these soils belong to the steppe type, namely chernozemolic soil (leached chernozem at the experimental field “Radmilovac” and chernozem at the experimental field “Rimski Šančevi”). The values of hygroscopic humidity (2.22–3.20%) confirm that the soil of both experimental fields corresponds to loam in terms of mechanical composition. Based on the division of soil according to density, the soil at the experimental field “Radmilovac” belongs to the slightly compacted arable land, while the soil at the experimental field “Rimski Šančevi” belongs to the strongly compacted arable land.

The results of determination of the chemical properties of the studied soil in two experimental fields at the 20 cm depth are presented in Table 4. The results of the investigation of the active acidity of the soil in the experimental field “Radmilovac” (pH in H_2_O) indicated that the soil at locations R1–R3 belongs to weakly acidic soils, while the soil at locations R4–R6 is neutral in terms of pH value. The values of pH in H_2_O for soil at the experimental field “Rimski Šančevi” indicated that this soil is sligthly to moderately alkaline. In the process of determining substitution acidity (pH in KCl), the corresponding soil chemical reactions are closer to neutral and slightly alkaline chemical reactions in the case of the experimental field “Radmilovac”, while for the experimental field “Rimski Šančevi”, the soil chemical reaction is neutral. The CaCO_3_ content values indicated that soil at the experimental field “Radmilovac” is weakly carbonated. In the experimental field “Rimski Šančevi”, the differences in the content of CaCO_3_ in the analyzed soil from different experimental plots were more pronounced, from non-carbonate without CaCO_3_, low carbonate, medium carbonate with 2–5% CaCO_3_, and up to carbonate with 5–10% CaCO_3_.

The content of the humus and the extracted organic carbon in the investigated soil samples at two experimental fields at a 20 cm depth are presented in Table 5. Based on the humus content, the analyzed soil of both experimental fields belongs to the class of low humus soil. The content of total organic carbon in the investigated soil of the “Radmilovac” varied from 0.23 to 0.42%, and in the soil of the “Rimski Šančevi”, from 0.18 to 0.33%. In Table 5, it can be seen that at the experimental field “Radmilovac”, the content of organic carbon in humic acids is higher than in fulvic acids, for all investigated plots. In the experimental field “Rimski Šančevi”,the same trend in the content of organic carbon in humic and fulvic acids does not exist for all investigated plots.

### 3.3. Linear Correlation Analysis and Analysis of Variance (ANOVA)

The linear correlation analysis presented in the Supplementary Material (Tables S1 and S2) was performed between the ^90^Sr activity concentration in the soil (labeled as ^90^Sr) and in the crops (labeled as ^90^Sr_root_ for the root of the plant and ^90^Sr_rest_ for the rest of the plant, including stem, leaf and grain) and the physico-chemical properties of the soil studied: coarse sand (CS), fine sand (FS), silt (SI), clay (CL), hygroscopic humidity (HH), density (DE), pH in H_2_O (pH_H2O_), pH in KCl (pH_KCl_), CaCO_3_ content (CC), humus content (HU), organic carbon content (OC), organic carbon content in humic acids (OC_H_) and organic carbon content in fulvic acids (OC_F_).

For the leached chernozem at the experimental field “Radmilovac”, significant positive correlations (*p* < 0.05) were found between the ^90^Sr activity concentration in the roots of the crop and the soil around the root system. The ^90^Sr activity concentration in the roots of the crops showed a significant correlation with the activity concentration in other plant organs, indicating an acropetal translocation of this radionuclide in the studied crops. The influence of soil properties on the variation in ^90^Sr activity concentrations in soil and crops showed a significant dependence between ^90^Sr activity concentration in crops and physical soil properties, i.e.*,* mechanical fractions (coarse and fine sand and clay), soil moisture and chemical properties of the soil (content of humus and total organic carbon, as well as humic and fulvic acids).

Significant positive correlations (*p* < 0.05) were found between the ^90^Sr activity concentration in the chernozem and the crops of the experimental field “Rimski Šančevi”. Significant correlations were found between the ^90^Sr activity concentration in the soil and the physico-chemical properties of the soil: fine sand, silt and clay content, moisture, density, humus content, total organic carbon content and pH in KCl.

Based on the linear correlation analysis carried out, it can be said that the physical and chemical properties of the soil have an influence on the behavior of the ^90^Sr radionuclide in the terrestrial ecosystem. The extent of the influence depends on the complexity of the interactions due to numerous processes occurring simultaneously in the soil and on the general conditions of the environment. It was also found that in the chernozem soil type, the physical and chemical properties of the soil have a much greater influence on the transfer of the radionuclide ^90^Sr into the soil and crops, which is influenced in particular by the content of clay and humus as well as soil moisture and pH. The behavior of this radionuclide in the soil profiles depends on the relative proportions of sand and clay in the soil, which is confirmed by the different soil textures of the studied fields; in “Radmilovac”, the soil texture was powdery loam, while in “Rimski Šančevi” it was predominantly loam. In clay soil, ^90^Sr migration can be influenced by the rate of infiltration in the clay layers, as this soil fraction adsorbs the radionuclides most strongly [10]. In addition to the clay content, soil moisture and humus content also have a major influence on the behavior of ^90^Sr in the soil, which is confirmed by a statistically significant correlation. This is consistent with the literature data that humic substances in clay soil facilitate interactions between soluble forms of anthropogenic radionuclides and soil particles, which enable their mobility in terrestrial ecosystems [37]. The reason for this is that fulvic acids can form complexes with cations in the soil that increase their solubility and mobility, which can also improve accessibility directly in the rhizosphere zone of crops. In addition, humic substances facilitate the interaction between the soluble forms of the radionuclide ^90^Sr and soil particles, which enables the migration of this radionuclide in the soil. The relationship between ^90^Sr and soil moisture variation, i.e., hygroscopic moisture content, could be indirectly related to the composition of clay, as the negative charge of most clay minerals attracts water molecules and thus generates hygroscopic moisture on their surface.

The one-factorial analysis of variance (ANOVA test) was applied to detect the presence of differences indicating a possible influence of the applied technologies in the cultivation of wheat, a crop present in both experimental fields during the study years. The analysis of variance of winter wheat depending on the experimental field showed that the significance level of *p* < 0.01 was obtained for ^90^Sr in the soil, as well as the root and the rest of the winter wheat. The existence of this difference may be due to differences in soil types and their properties, and the cultivation technology and agrotechnical measures applied also play a role. The results suggest that the influence of the application of appropriate agrotechnical measures in the experimental fields could be monitored and controlled using certain parameters.

### 3.4. Calculated Values of the Transfer Factor in the Agricultural Soil–Crop System

Based on the measured activity concentration of ^90^Sr in the cultivated soil and crops, the transfer factor was calculated as a parameter for radiation safety. The results of the calculated transfer factor are presented in Table 6 for both experimental fields. Since the activity concentrations of ^90^Sr in the maize grain of all the maize samples tested in the experimental field “Rimski Šančevi” were below the detection limit, the results of the soil–maize grain transfer factor for this experimental field are not presented. The maximum value of the calculated parameter was obtained for the soybean samples, which belongs to the legumes. The fact is that legumes have a higher affinity for the uptake of ^90^Sr from the soil than cereals and industrial plants.

**Figure 2:**
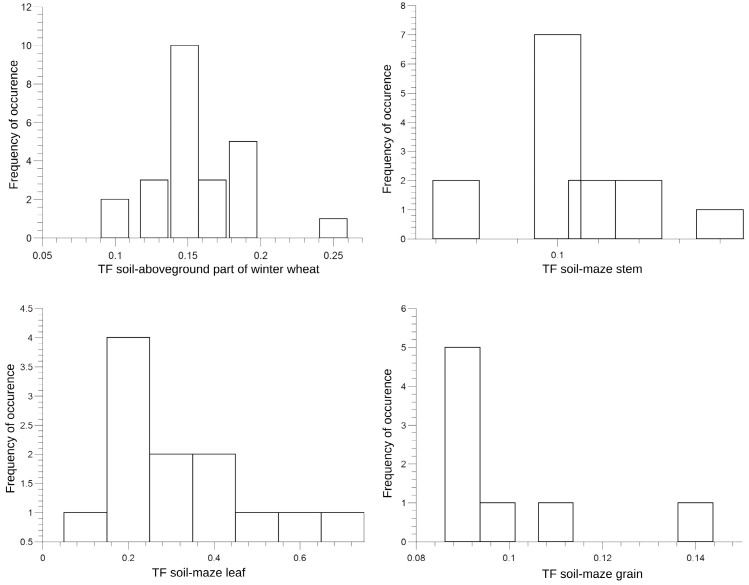
Bar-diagram of the frequency of values of the ratio between soil and crop activity concentration.

Comparing the values of the transfer factor for winter wheat and maize analyzed in both experimental fields, the frequency of occurrence of the corresponding value can be determined. This is shown in Figure 2.

It can be seen that the expressed maximum occurs for a certain value, which corresponds to the value for the transfer factor of the corresponding plant organ for the largest number of analyzed samples. The relative frequency of the maximum value of the transfer factor for the plant organs of both crops is about 40%. The results of the frequency of occurrence of a given value of the soil–crop transfer factor for the radionuclide ^90^Sr indicate that this radionuclide is the most absorbed in the root of the plant. The presented results confirm the fact that the important mechanism of accumulation of long-lived radionuclides in plants is absorption from the soil [38,39]. The values of the soil–crop transfer factor for the radionuclide ^90^Sr presented in this study are in accordance with the results reported in the world literature [12,14,15,38,40,41]. In general, the overall results of this study are in line with the results reported in a review paper by Burger and Lichtscheidl [9]. Considering the fact that this parameter is a relevant bioindicator for checking the radiation exposure of the soil–plant system, the obtained results indicate that the crops grown in the area of the investigated experimental fields in the Republic of Serbia are not significantly contaminated with this radionuclide.

### 3.5. Comparison of the Results of the Applied Mathematical Model and the Experimentally Determined Values of the Transfer Factor

For the modeling, the values of the input parameters described by Equation (5), K_c_ (^90^Sr^2+^/Ca^2+^) and α_ex_, for the respective soil types, were taken from the study reported by Sysoeva et al. [42], while the value of the CEC parameter was taken from Dragović et al. [43]. Krogh et al. [44] have shown that the value for the physico-chemical parameter CEC can be calculated by the pedotransfer function if the values for soil organic matter and clay content are known. For the experimental design of this study, both values were calculated using the pedotransfer function according to Krogh et al. [44] and the values obtained were in agreement with those reported in Dragović et al. [43]. For the leached chernozem soil type of the experimental field “Radmilovac”, the following values of the input parameters were used: 2.1 for K_c_ (^90^Sr^2+^/Ca^2+^), 0.7 for α_ex_ and 30 cmol/kg for CEC, while for the chernozem soil type of the experimental field “Rimski Šančevi” the values were 1.9, 0.7 and 32 cmol/kg, respectively. Table 7 shows the comparison of the results of the applied mathematical model with the experimentally determined values of the transfer factor [7].

The crops grown on the experimental field “Radmilovac” (site code R1–R6) during the study period (2013 to 2015) were winter wheat and maize. The following crops were cultivated on the experimental field “Rimski Šančevi” (site code NS1–NS6) during investigated period (2013 to 2015 year): winter wheat monoculture (site code NS1), maize monoculture (site code NS2), soybean monoculture (site code NS3) and rapeseed (site code NS10). The crop rotation was shown at the other sites of the experimental field “Rimski Šančevi”. The deviation of the modeling results from the experimentally determined values is due to the fact that the model assumes that all ion exchange centers in the soil are occupied by calcium ions (i.e.*,* that the concentration of exchangeable calcium is equal to the CEC parameter). In addition, the model assumes that the crop only takes up ^90^Sr via the root system, which is not the only form of uptake for all plants. Some crops are characterized by a dual mechanism by which the crop can take up nutrients (via the root and also via the leaves), resulting in a relatively high concentration of ^90^Sr activity in individual plant organs.

When comparing the experimentally determined values of the transfer factor with the values determined using the uptake model proposed by Maskalchuk et al. [7], relatively good agreement was found. This indicates that the model used here has the possibility of application in the prediction of ^90^Sr migration in the soil–soil solution–crop system under microsite-specific conditions of selected agricultural fields. In addition, this study confirmed that a technical approach for designing and conducting an experiment to determine the uptake of selected artificial radionuclide was successfully carried out and mathematically supported for the selected part of the agroecosystem.

## 4. Conclusions

The conducted research focused on the content and accumulation of ^90^Sr in cultivated soil and in parts of cultivated crops under corresponding conditions in two experimental fields in the Republic of Serbia. The analysis of ^90^Sr accumulation for the chernozem soil type and the corresponding crops clearly showed a significant uptake of the ^90^Sr.

A linear correlation analysis was used to determine the most important physico–chemical, chemical and soil parameters that influence the transfer of ^90^Sr in the agroecosystem. The results of the analysis showed that there are statistically significant correlations between the ^90^Sr activity concentration and the physico–chemical properties of the soil at the studied sites, with significance levels of *p* < 0.001; 0.01 and 0.05. The analysis of the correlations allows us to conclude that the humus content, i.e., its component fulvic acid, is a significant factor influencing the sorption of the radionuclide ^90^Sr in the arable soil layer. The distribution of ^90^Sr and its transfer in arable soil depend to a considerable extent on the content of the clay fraction in the soil, as this is characterized by the ability to exchange cations. The results of the correlation analysis indicated a relationship between the activity concentration of ^90^Sr and its behavior in hygroscopic soil moisture, which is most likely an indirect link with the soil fraction with fine-structure clay.

The obtained experimental results were processed with the help of a mathematical model. The results of modeling the migration of ^90^Sr from cultivated soil to the aerial part of crops showed relatively good agreement with the calculated values of the soil–crop transfer factor based on the experimentally determined values. This indicates a possibility that the model proposed by Maskalchuk et al. can be applied in experimental conditions. However, this paper is the only paper referring to the mentioned study type, and there are not any published papers that have experimentally investigated the present model. Since TF has no scientific meaning and is simply to indicate the degree of absorption of the specific element (^90^Sr radionuclide in this case) from soil by a plant, another conclusion would be that this study experimentally supports a scientific meaning of TF for ^90^Sr.

The data from this study provide an initial database for predicting fluctuations in artificial radioactivity levels in the agroecosystem. These results improved our current understanding and knowledge on ^90^Sr levels in soil and plants, transfer pathways and trends in different parts of the agroecosystem, and we aim to continue the detailed radioecological research related to ^90^Sr. Such experiments and research should be continued, evaluated and extended to other parts of the terrestrial ecosystem that are directly or indirectly impacted by radioactive waste so that proper strategies can be put in place to minimize the negative impacts of radioactive materials on our health of soil, plants, animal, humans and ecosystems.

## Figures and Tables

**Table 1:** The activity concentration of ^90^Sr in cultivated soil samples over three years.

Location Code/Year	A_Sr-90_ (Bq/kg)		
	2013	2014	2015
	Experimental field “Radmilovac”		
R1	2.98 ± 0.59	3.16 ± 0.62	1.81 ± 0.37
R2	2.68 ± 0.54	2.67 ± 0.56	2.19 ± 0.45
R3	2.49 ± 0.51	2.73 ± 0.56	1.97 ± 0.41
R4	3.74 ± 0.73	2.48 ± 0.47	1.92 ± 0.35
R5	3.63 ± 0.76	3.14 ± 0.64	2.36 ± 0.48
R6	3.51 ± 0.71	2.75 ± 0.58	2.09 ± 0.42
	Experimental field “Rimski Šančevi”		
NS1	2.25 ± 0.45	1.88 ± 0.38	1.72 ± 0.33
NS2	2.13 ± 0.41	1.55 ± 0.32	1.48 ± 0.29
NS3	2.56 ± 0.53	1.78 ± 0.39	1.64 ± 0.32
NS4	3.17 ± 0.61	2.72 ± 0.53	2.67 ± 0.58
NS5	2.83 ± 0.54	1.99 ± 0.39	1.67 ± 0.32
NS6	3.01 ± 0.59	2.45 ± 0.47	2.09 ± 0.42
NS7	2.45 ± 0.49	1.64 ± 0.33	1.54 ± 0.32
NS8	2.32 ± 0.44	1.87 ± 0.37	1.27 ± 0.26
NS9	2.69 ± 0.54	2.25 ± 0.43	1.98 ± 0.41
NS10	2.29 ± 0.43	1.42 ± 0.27	1.24 ± 0.25

**Table 2:** The activity concentration of ^90^Sr in cultivated crop samples (ww—winter wheat; m—maize; s—soybean; r—rapeseed) over three years.

	A_Sr-90_ (Bq/kg)					
	Experimental field “Radmilovac”					
**Year/crop type**	**2013 (Winter wheat)**					
**Part of crop/** **location code**	**R1**	**R2**	**R3**	**R4**	**R5**	**R6**
Root	1.72 ± 0.26	1.50 ± 0.23	1.37 ± 0.21	1.60 ± 0.24	1.05 ± 0.16	1.13 ± 0.17
Rest of plant	0.51 ± 0.07	0.46 ± 0.07	0.45 ± 0.07	0.53 ± 0.08	0.46 ± 0.07	0.49 ± 0.07
**Year/crop type**	**2014 (Maize)**					
Root	1.38 ± 0.22	1.32 ± 0.21	0.93 ± 0.14	1.67 ± 0.25	1.38 ± 0.22	1.48 ± 0.22
Stem	0.36 ± 0.05	0.29 ± 0.04	0.27 ± 0.04	0.44 ± 0.06	0.34 ± 0.05	0.38 ± 0.06
Leaf	0.68 ± 0.11	0.64 ± 0.09	0.58 ± 0.08	0.80 ± 0.12	0.54 ± 0.07	0.60 ± 0.09
Grain	0.30 ± 0.04	0.27 ± 0.04	0.25 ± 0.04	0.35 ± 0.05	0.29 ± 0.04	0.31 ± 0.04
**Year/crop type**	**2015 (Winter wheat)**					
Root	1.37 ± 0.21	1.24 ± 0.19	1.14 ± 0.17	1.47 ± 0.22	0.77 ± 0.11	0.79 ± 0.15
Rest of plant	0.33 ± 0.05	0.31 ± 0.05	0.28 ± 0.04	0.36 ± 0.06	0.26 ± 0.04	0.28 ± 0.05
	Experimental field “Rimski Šančevi”					
**Year**	**2013**					
**Part of crop/location code**	**NS1 *^ww^***	**NS4 *^ww^***	**NS5 *^ww^***	**NS9 *^ww^***		**NS10 *^r^***
Root	0.74 ± 0.11	0.96 ± 0.14	0.84 ± 0.12	0.93 ± 0.14		0.52 ± 0.08
Rest of plant	0.39 ± 0.06	0.45 ± 0.06	0.41 ± 0.06	0.43 ± 0.07		0.23 ± 0.03
	**NS2 *^m^***	**NS3 *^s^***	**NS6 *^s^***	**NS7 *^s^***		**NS8 *^s^***
Root	1.15 ± 0.18	1.24 ± 0.18	1.31 ± 0.21	1.03 ± 0.15		1.17 ± 0.18
Stem	0.21 ± 0.03	0.82 ± 0.12	0.64 ± 0.09	0.57 ± 0.09		0.72 ± 0.11
Leaf	0.95 ± 0.14	0.98 ± 0.14	1.17 ± 0.18	0.89 ± 0.13		0.93 ± 0.14
Grain	<0.14	0.27 ± 0.04	0.38 ± 0.06	0.31 ± 0.05		0.24 ± 0.04
**Year**	**2014**					
	**NS1 *^ww^***	**NS6 *^ww^***	**NS7 *^ww^***	**NS8 *^ww^***		**NS10 *^r^***
Root	0.74 ± 0.11	0.79 ± 0.12	0.86 ± 0.13	0.81 ± 0.11		0.48 ± 0.07
Rest of plant	0.35 ± 0.05	0.36 ± 0.05	0.42 ± 0.06	0.37 ± 0.05		0.23 ± 0.03
	**NS2 *^m^***	**NS3 *^s^***	**NS4 *^m^***	**NS5 *^m^***		**NS9 *^s^***
Root	1.17 ± 0.18	1.20 ± 0.18	1.13 ± 0.17	1.07 ± 0.16		1.27 ± 0.19
Stem	0.19 ± 0.03	0.75 ± 0.11	0.19 ± 0.03	0.19 ± 0.03		0.66 ± 0.09
Leaf	0.82 ± 0.12	0.94 ± 0.14	0.81 ± 0.12	0.91 ± 0.14		1.12 ± 0.17
Grain	<0.10	0.22 ± 0.03	<0.11	<0.08		0.20 ± 0.03
**Year**	**2015**					
	**NS1 *^ww^***	**NS4 *^ww^***	**NS5 *^ww^***	**NS9 *^ww^***		**NS10 *^r^***
Root	0.65 ± 0.09	0.88 ± 0.13	0.75 ± 0.11	0.81 ± 0.11		0.47 ± 0.07
Rest of plant	0.26 ± 0.04	0.32 ± 0.05	0.25 ± 0.04	0.21 ± 0.03		0.19 ± 0.03
	**NS2 *^m^***	**NS3 *^s^***	**NS6 *^m^***	**NS7 *^m^***		**NS8 *^m^***
Root	1.15 ± 0.17	0.95 ± 0.14	1.08 ± 0.16	1.18 ± 0.17		1.03 ± 0.15
Stem	0.13 ± 0.02	0.78 ± 0.12	0.06 ± 0.01	0.08 ± 0.01		0.14 ± 0.02
Leaf	1.07 ± 0.16	0.84 ± 0.13	0.83 ± 0.12	0.82 ± 0.12		0.78 ± 0.12
Grain	<0.11	0.41 ± 0.08	<0.05	<0.06		<0.03

**Table 3:** The physical properties of investigated soil samples (SCL—silty clay loam, CL—clay loam, SaCL—sandy clay loam).

Location Code	Mechanical Composition (%)				Texture [36]	Hygroscopic Humidity (%)	Density (g/cm^3^)
	Coarse Sand	Fine Sand	Silt	Clay			
R1	3.73	2.95	62.26	31.06	SCL	2.55	1.29
R2	4.12	3.27	63.42	29.19	SCL	2.62	1.33
R3	2.87	3.13	61.25	32.75	SCL	2.42	1.31
R4	9.23	7.11	56.31	27.35	SCL	2.84	1.30
R5	8.41	5.02	53.83	32.74	SCL	2.37	1.27
R6	7.97	6.28	52.48	33.27	SCL	2.22	1.25
Average value	6.06	4.63	58.26	31.06	/	2.50	1.29
NS1	12.99	16.59	33.47	36.95	CL	3.07	1.33
NS2	8.56	21.60	32.84	37.00	CL	3.15	1.30
NS3	15.46	15.84	35.08	33.62	CL	3.20	1.31
NS4	12.68	24.53	34.85	27.95	SCL	2.65	1.35
NS5	12.01	20.45	33.42	34.12	CL	2.94	1.32
NS6	10.04	53.89	9.04	27.05	SaCL	3.17	1.39
NS7	9.57	20.34	34.23	35.86	CL	3.10	1.36
NS8	8.32	22.22	34.93	34.56	CL	3.07	1.37
NS9	11.42	25.54	36.15	26.89	CL	2.71	1.34
NS10	11.49	6.88	44.09	37.54	SCL	3.15	1.38
Average value	11.25	22.79	32.81	33.15	/	3.02	1.34

**Table 4:** The chemical properties of investigated soil samples.

Location Code	pH in H_2_O	pH in KCl	CaCO_3_ (%)
R1	6.23	6.77	1.40
R2	6.38	6.75	1.28
R3	6.56	7.00	1.52
R4	7.00	7.56	1.20
R5	7.12	7.87	1.35
R6	7.35	7.92	1.64
Average value	6.77	7.31	1.40
NS1	7.61	6.54	0.70
NS2	7.43	6.58	/
NS3	7.80	6.93	2.52
NS4	7.76	7.15	4.21
NS5	7.80	7.02	0.24
NS6	7.58	6.73	/
NS7	7.68	6.86	1.26
NS8	7.99	7.12	3.93
NS9	7.67	7.12	6.88
NS10	7.69	6.98	1.26
Average value	7.70	6.90	2.10

**Table 5:** Content of the humus and the extracted organic carbon in investigated soil samples.

Location Code	Humus (%)	Total Organic Carbon (%)	Organic Carbon in Humic Acid (%)	Organic Carbon in Fulvic Acid (%)
R1	2.01	0.42	0.24	0.18
R2	2.37	0.38	0.22	0.16
R3	2.47	0.34	0.20	0.14
R4	2.08	0.40	0.27	0.13
R5	2.12	0.26	0.18	0.08
R6	2.63	0.23	0.14	0.09
Average value	2.28	0.34	0.21	0.13
NS1	3.03	0.25	0.13	0.12
NS2	2.80	0.26	0.15	0.11
NS3	2.86	0.24	0.18	0.06
NS4	2.29	0.18	0.05	0.13
NS5	2.66	0.28	0.14	0.14
NS6	2.93	0.24	0.11	0.13
NS7	2.97	0.33	0.11	0.22
NS8	2.93	0.31	0.17	0.14
NS9	2.52	0.26	0.19	0.07
NS10	2.36	0.25	0.12	0.13
Average value	2.74	0.26	0.14	0.12

**Table 6:** The ^90^Sr transfer factor for cultivated soil–crops system (ww—winter wheat; m—maize; s—soybean; r—rapeseed).

	TF					
	Experimental field “Radmilovac”					
**Year/crop type**	**2013 (Winter wheat)**					
**Part of crop/location code**	**R1**	**R2**	**R3**	**R4**	**R5**	**R6**
Root	0.58	0.56	0.55	0.43	0.29	0.32
Rest of plant	0.17	0.17	0.18	0.14	0.13	0.14
**Year/crop type**	**2014 (Maize)**					
Root	0.44	0.49	0.34	0.67	0.44	0.54
Stem	0.11	0.11	0.10	0.18	0.11	0.14
Leaf	0.22	0.24	0.21	0.32	0.17	0.22
Grain	0.10	0.10	0.09	0.14	0.09	0.11
**Year/crop type**	**2015 (Winter wheat)**					
Root	0.76	0.57	0.58	0.77	0.33	0.38
Rest of plant	0.18	0.14	0.14	0.19	0.11	0.13
	Experimental field “Rimski Šančevi”					
**Year**	**2013**					
**Part of crop/location code**	**NS1*^ww^***	**NS4*^ww^***	**NS5*^ww^***	**NS9*^ww^***		**NS10*^r^***
Root	0.33	0.30	0.30	0.35		0.23
Rest of plant	0.17	0.14	0.14	0.16		0.10
	**NS2*^m^***	**NS3*^s^***	**NS6*^s^***	**NS7*^s^***		**NS8*^s^***
Root	0.54	0.48	0.44	0.42		0.50
Stem	0.10	0.32	0.21	0.23		0.31
Leaf	0.45	0.38	0.39	0.36		0.40
Grain	/	0.11	0.13	0.13		0.10
**Year**	**2014**					
	**NS1*^ww^***	**NS6*^ww^***	**NS7*^ww^***	**NS8*^ww^***		**NS10*^r^***
Root	0.39	0.32	0.52	0.43		0.34
Rest of plant	0.19	0.15	0.26	0.20		0.16
	**NS2*^m^***	**NS3*^s^***	**NS4*^m^***	**NS5*^m^***		**NS9*^s^***
Root	0.76	0.67	0.42	0.54		0.56
Stem	0.12	0.42	0.07	0.10		0.29
Leaf	0.53	0.53	0.30	0.46		0.50
Grain	/	0.12	/	/		0.09
**Year**	**2015**					
	**NS1*^ww^***	**NS4*^ww^***	**NS5*^ww^***	**NS9*^ww^***		**NS10*^r^***
Root	0.38	0.33	0.45	0.41		0.38
Rest of plant	0.15	0.12	0.15	0.11		0.15
	**NS2*^m^***	**NS3*^s^***	**NS6*^m^***	**NS7*^m^***		**NS8*^m^***
Root	0.78	0.58	0.52	0.77		0.81
Stem	0.09	0.48	0.03	0.05		0.11
Leaf	0.72	0.51	0.40	0.53		0.61
Grain	/	0.25	/	/		/

**Table 7:** Comparison of the experimentally determined values of the transfer factor for ^90^Sr (TF_exp_) and the values determined with the proposed model (TF_mod_).

Year	2013		2014		2015		Average Value of TF_mod_/TF_exp_Ratio
Location Code	TF_mod_	TF_exp_	TF_mod_	TF_exp_	TF_mod_	TF_exp_	
R1	0.13	0.17	0.12	0.11	0.18	0.18	0.94
R2	0.14	0.17	0.10	0.11	0.11	0.14	0.84
R3	0.15	0.18	0.13	0.10	0.11	0.14	0.97
R4	0.15	0.14	0.12	0.18	0.14	0.19	0.82
R5	0.18	0.13	0.12	0.11	0.15	0.11	1.28
R6	0.20	0.14	0.12	0.14	0.15	0.13	1.12
NS1	0.25	0.17	0.22	0.19	0.19	0.15	1.29
NS2	0.09	0.10	0.08	0.12	0.05	0.09	0.70
NS3	0.31	0.32	0.39	0.42	0.39	0.30	1.06
NS4	0.22	0.14	0.08	0.07	0.17	0.12	1.40
NS5	0.23	0.15	0.08	0.10	0.16	0.15	1.17
NS6	0.23	0.21	0.22	0.15	0.03	0.03	1.15
NS7	0.26	0.23	0.23	0.26	0.03	0.05	0.88
NS8	0.29	0.31	0.22	0.20	0.06	0.11	0.87
NS9	0.22	0.16	0.24	0.25	0.12	0.11	1.16
NS10	0.11	0.10	0.22	0.16	0.19	0.15	1.24

## Data Availability

Data are contained within the article and Supplementary Materials.

## Supplementary Materials

The following supporting information can be downloaded at: https://www.mdpi.com/article/10.3390/plants13131798/s1, Table S1: Correlation coefficients (experimental field “Radmilovac”); significance level: * *p* < 0.05, ** *p* < 0.01, *** *p* < 0.001; Table S2: Correlation coefficients (experimental field “Rimski Šančevi”); significance level: * *p* < 0.05, ** *p* < 0.01, *** *p* < 0.001.
