# Supplementary material for: Application and Experimental Substantiation of the Radioecological Model for Prediction in Behavior 90Sr in Cultivated Soil-Crop System: A Case Study of Two Experimental Agricultural Fields
Source: Plants (Basel). 2024 Jun 29;13(13):1798. doi: 10.3390/plants13131798 (PMC11243939; doi:10.3390/plants13131798)
Supplement: Supplementary file 1 [file plants-13-01798-s001.zip › Table S1 sm corrected.pdf]

	Year	^{90}Sr	^{90}Sr$_{root}$	^{90}Sr$_{rest}$	CS	FS	SI	CL	HH	DE	pH$_{H2O}$	pH$_{KCl}$	CC	HU	OC	OC$_{H}$	OC$_{F}$
	2013	1	-0.78					**0.91****							0.86		
**^{90}Sr**	2014	1	**0.91*****					**0.92****						**0.93****			**0.91****
	2015	1						-0.81	0.89					0.88			0.87
	2013		1					**0.93****	0.89					0.77	**0.97****	0.86	
**^{90}Sr$_{root}$**	2014		1	**0.93****	0.79			-0.89									
	2015		1	**0.91****				-0.81	0.89						**0.97*****	**0.94****	0.82
	2013			1													
**^{90}Sr$_{rest}$**	2014			1	0.78	0.82		-0.88	0.86							0.81	
	2015			1				-0.88	0.88						0.82	0.87	
	2013				1	**0.94****	-0.87				0.86	0.88					
**CS**	2014				1	**0.94****	-0.87				0.86	0.88					
	2015				1	**0.94****	-0.87				0.86	0.88					
	2013					1	-0.82				0.86	0.83					
**FS**	2014					1	-0.82				0.86	0.83					
	2015					1	-0.82				0.86	0.83					
	2013						1			0.85	**-0.98*****	**-0.99*****					**0.92****
**SI**	2014						1			0.85	**-0.98*****	**-0.99*****			0.78		**0.92****
	2015						1			0.85	**-0.98*****	**-0.99*****			0.78		**0.92****
	2013							1					0.85	**-0.96*****		-0.86	
**CL**	2014							1	**-0.96****				0.85			-0.86	
	2015							1	**-0.96****				0.85				-0.86
	2013								1								
**HH**	2014								1				-0.88		0.84	**0.96****	
	2015								1				-0.88		0.84		**0.96****
	2013									1		-0.8					
**DE**	2014									1		-0.8					
	2015									1		-0.8					
	2013										1	**0.98*****			0.79		**-0.94****
**pH$_{H2O}$**	2014										1	**0.98*****			-0.79		**-0.94****
	2015										1	0.98			-0.79		**-0.94****
	2013											1			-0.78		**-0.95****
**pH$_{KCl}$**	2014											1			-0.78		**-0.95****
	2015											1			-0.78		**-0.95****
	2013												1	-0.88		-0.81	
**CC**	2014												1			-0.81	
	2015												1			-0.81	
	2013													1	0.84	**0.96****	
**HU**	2014													1			
	2015													1			
	2013														1	**0.93****	**0.9****
**OC**	2014														1	**0.93****	**0.9****

	2015	1	**0.93****	**0.9****
**OC_H**	2013		1	
	2014		1	
	2015		1	
**OC_F**	2013			1
	2014			1
	2015			1

Table 1sm: Correlation coefficients (experimental field "Radmilovac"); significance level: *p < 0.05, **p < 0.01, ***p < 0.001
