# Supplementary material for: Application and Experimental Substantiation of the Radioecological Model for Prediction in Behavior 90Sr in Cultivated Soil-Crop System: A Case Study of Two Experimental Agricultural Fields
Source: Plants (Basel). 2024 Jun 29;13(13):1798. doi: 10.3390/plants13131798 (PMC11243939; doi:10.3390/plants13131798)
Supplement: Supplementary file 1 [file plants-13-01798-s001.zip › Table S2 sm corrected.pdf]

	Year	^{90}Sr	^{90}Sr$_{root}$	^{90}Sr$_{rest}$	CS	FS	SI	CL	HH	DE	pH$_{H2O}$	pH$_{KCl}$	CC	HU	OC	OC$_{H}$	OC$_{F}$
**^{90}Sr**	2013	1	0.85	0.89					-0.80					**-0.94****			**-0.93****
	2014	1	0.85					-0.8	**-0.91****	0.88				**-0.91****	-0.86		**-0.91****
	2015	1	0.85					-0.8	**-0.91****	0.88				**-0.91****	-0.86		**-0.91****
**^{90}Sr$_{root}$**	2013		1	**0.98*****		**0.97*****		**-0.96****	**-0.99*****			0.88		**-0.98*****			
	2014		1	**0.93****							0.82						
	2015		1	**0.93****							0.82						
**^{90}Sr$_{rest}$**	2013			1		**0.91****		**0.91****	**-0.98*****					**-0.98*****			
	2014			1													
	2015			1													
**CS**	2013				1												
	2014				1												
	2015				1												
**FS**	2013					1		**-0.99*****	**-0.97*****			**0.92****		**-0.91****			
	2014					1	0.87	**-0.99*****	**-0.97*****				0.87	**-0.91*****			
	2015					1	0.87	**-0.99*****	**-0.97*****				0.87	**-0.91*****			
**SI**	2013						1	**-0.92****					**0.99*****				
	2014						1	**-0.92****	-0.82				**0.99*****				-0.83
	2015						1	**-0.92****	-0.82				**0.99*****				-0.83
**CL**	2013							1					**-0.92****	0.88			
	2014							1		**0.98*****		-0.86	**-0.92****	0.88			
	2015							1		**0.98*****		-0.86	**-0.92****	0.88			
**HH**	2013								1					**0.95****			
	2014								1	-0.81		-0.88	-0.83	**0.95****			
	2015								1	-0.81		-0.88	-0.83	**0.95****			
**DE**	2013									1							
	2014									1					-0.86		
	2015									1					-0.86		
**pH$_{H2O}$**	2013										1						
	2014										1						
	2015										1						
**pH$_{KCl}$**	2013											1		**-0.94****			
	2014											1		**-0.94****			
	2015											1		**-0.94****			
**CC**	2013												1				
	2014												1				-0.81
	2015												1				-0.81
**HU**	2013													1			
	2014													1			
	2015													1			
**OC**	2013														1		
	2014														1	0.85	

	2015	1	0.85	
	2013		1	
**OC_H**	2014		1	
	2015		1	
	2013			1
**OC_F**	2014			1
	2015			1

Table 2sm: Correlation coefficients (experimental field "Rimski Šančevi"); significance level: *p < 0.05, **p < 0.01, ***p < 0.001
